# Bridging the Health Data Divide

**DOI:** 10.2196/jmir.6400

**Published:** 2016-12-20

**Authors:** Leo Anthony Celi, Guido Davidzon, Alistair EW Johnson, Matthieu Komorowski, Dominic C Marshall, Sunil S Nair, Colin T Phillips, Tom J Pollard, Jesse D Raffa, Justin D Salciccioli, Francisco Muge Salgueiro, David J Stone

**Affiliations:** ^1^ MIT Critical Data Cambridge, MA United States

**Keywords:** electronic health records, machine learning, health care policy, medical education, collaboration

## Abstract

Fundamental quality, safety, and cost problems have not been resolved by the increasing digitization of health care. This digitization has progressed alongside the presence of a persistent divide between clinicians, the domain experts, and the technical experts, such as data scientists. The disconnect between clinicians and data scientists translates into a waste of research and health care resources, slow uptake of innovations, and poorer outcomes than are desirable and achievable. The divide can be narrowed by creating a culture of collaboration between these two disciplines, exemplified by events such as datathons. However, in order to more fully and meaningfully bridge the divide, the infrastructure of medical education, publication, and funding processes must evolve to support and enhance a learning health care system.

## Introduction

The US Agency for Healthcare Research and Quality (AHRQ) was established in 1989 in response to an Institute of Medicine (now the National Academy of Medicine) report that pointed out “escalating healthcare costs, wide variations in medical practice patterns, and evidence that some health services are of little or no value” [1]. More than 25 years later, there has been surprisingly, perhaps even shockingly, little progress in these three areas. Quality of care, as would be reflected by the universal provision of standardized, evidence-based, and truly indicated care, has not improved to the degree one would have hoped. Similarly, while medical safety and errors have also come increasingly into the awareness of the medical system over that 25-year time period [2], advances in these areas have been slow, hard won, and unsupported by the kinds of smart, data-driven engineering designs that have gone into other domains.

**Figure 1:**
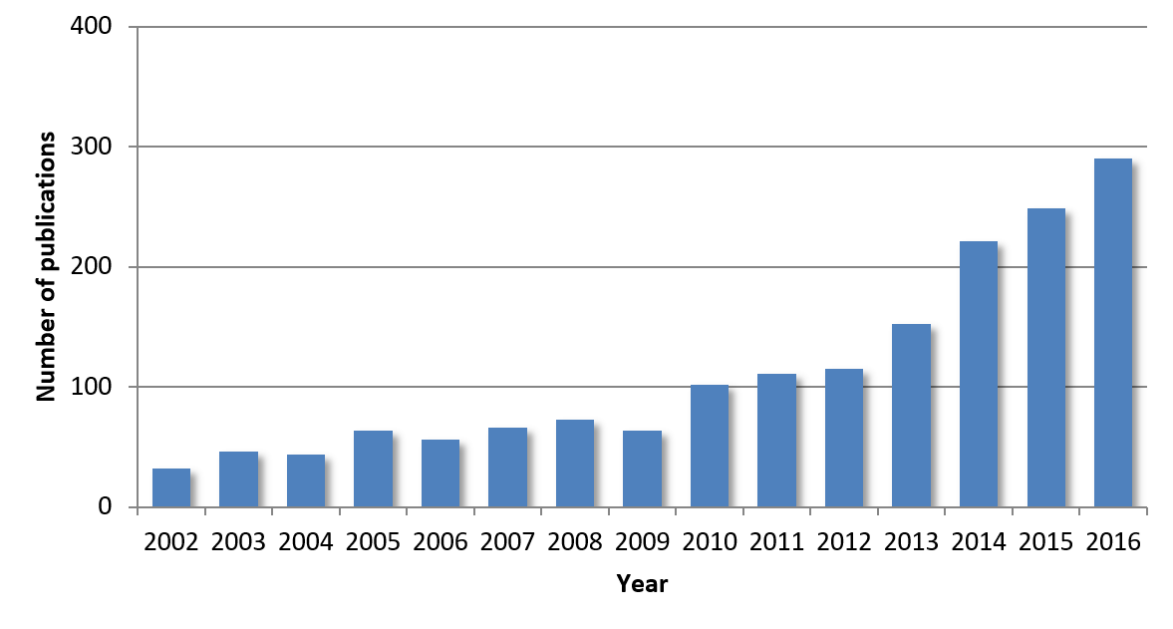
PubMed search results for ("sepsis"[All Fields] OR "septic"[All Fields]) AND ("machine learning"[All Fields] OR "data analysis"[All Fields] OR "data science"[All Fields] OR "engineering"[All Fields] OR "computing"[All Fields] OR "prediction"[All Fields]) AND ("2002/01/01"[PDAT] : "2016/12/31"[PDAT]).

Recent increases in computing power and data storage have resulted in an entirely new field involving the analysis of digitally archived information to acquire new knowledge: data science. While quality of care has largely been defined by clinical trials and expensive prospective studies, the application of data science to the clinical domain has the opportunity to dramatically increase the speed at which knowledge is generated and the breadth of questions that can be answered. The answers given are of particular interest when the research would otherwise be impractical, one such example being the comparison of an augmented treatment with a small effect size (normally requiring a prohibitively large prospective cohort). Data science has the opportunity to inform clinical decision making more directly as well, forecasting the occurrence of relevant clinical phenomena such as physiologic deterioration, diagnosis, medication adherence, or organ rejection. Machine learning, a field that was nascent in 1989 when the AHRQ was established, has now become ubiquitous and informs aspects of our everyday life from search queries to optimal routes. In the decade preceding the publication of the Surviving Sepsis Campaign guidelines [3], there was a significant increase in the number of publications that evaluated the use of machine learning in decision support and prognostication in sepsis (Figure 1).

The interest in applying machine learning to clinical practice is increasing, yet the practical application of these techniques has been less than desirable. Practitioners continue to make determinations in a technically unsupported and unmonitored manner due to a lack of high-quality evidence or tools to support most day-to-day decisions, and as a result the rate of diagnostic errors by individual practitioners is unacceptably high [4].

There is a persistent gap between the clinicians required to understand the clinical relevance of the data and the data scientists who are critical to extracting useable information from the increasing amount of health care data that are being generated. In this paper, we focus on the divide between the data science and health care silos, and posit that the lack of integration is the primary barrier to a data revolution in health care. We first discuss published literature that supports the existence of this divide, and then we present recommendations on how to bridge the gap between practicing clinicians and data scientists.

## The Divide

In both the practice and research arenas, there exists a divide between scientists and engineers on the one hand and, on the other, the clinicians who are most familiar with the exigencies and uncertainties that define and constrain the practice of medicine. There are several reasons for the development of this situation. Until recently, there have simply been very limited sources of data available to perform research such as the determination of comparative effectiveness, cost analysis, and the elucidation of treatment effect heterogeneity. Further, there has been a lack of strong motivation (ie, little, to no, to negative financial incentives; a lack of substantiating research; and strong individual and industry inertia and resistance) to reduce wasteful and sometimes harmful practice variation, or avoid (generally well-reimbursed) health services that are of little or no value.

In 2012, Kiri Wagstaff of California Institute of Technology’s Jet Propulsion Laboratory published an insightful article entitled “Machine learning that matters,” which pointed out how disconnected much of machine learning research is from important (and real) problems in society, including those of health care [5]. With the aim of refocusing efforts on topics that matter, Wagstaff proposed several “Impact Challenges” that tie machine learning to real-world outcomes, such as saving a human life or making significant financial savings through improved decision making. In a 2014 special issue of the journal *Machine Learning* [6], Rudin and Wagstaff explored the connection between machine learning research and its broader-world applications in more detail, and explicitly emphasized the importance of interdisciplinary collaboration for development of impactful research. While there is a clear need for machine learning in a variety of practical applications, the authors suggested that lack of enthusiasm in top venues to promote such work creates a “contradictory situation” that holds it back. Such situations serve to reinforce the health data divide.

## Bridging the Divide

### Recommendation 1: Collaboration

Given the definition of the problem, the most obvious solution, and yet the most challenging one, is encouraging collaboration between data scientists and clinicians. The incorporation of statistics into clinical research in the past 50 years and the rise of the biostatistician can act as a template from which the community can learn. The role of a biostatistician in biomedical research has become collaborative over time, at least partly due to the history and traditions of the discipline. An important event in this history in the United States was the passage of the 1962 Kefauver-Harris amendments to the Federal Food, Drug, and Cosmetic Act, which established in a preliminary form the method by which drugs are evaluated by the Food and Drug Administration today [7]. The act itself mandated proof of efficacy for new drugs, which had not been required before its signing into law. This piece of legislation and the ensuing events in the following two decades brought statisticians into close contact with clinician investigators, who now needed the statistician’s expertise to design, analyze, and report their study, and effectively established the tradition of collaboration between biostatisticians and clinician scientists seen to this day.

Can this process be accelerated between data scientists and those immersed in the practice of medicine? At the novel Icahn Institute for Genomics and Multiscale Biology at Mount Sinai Health System in New York, more than 300 people were hired to staff the new institute, with backgrounds across hardware design, big data computing, gene sequencing, and bioinformatics [8]. By linking this talent with disease centers within Mount Sinai, and using the tools of machine learning and predictive modeling (elements of big data), scientists have already published on inflammatory models in common-variant Alzheimer disease [9] and are taking a closer look at one of the most complex and biodiverse cell populations in the human body, the gut microbiome, which may be responsible for far more of the body’s homeostasis than previously realized [10].

Bridging the divide may be facilitated by instilling researchers with a greater appreciation of the benefits offered through collaboration with colleagues of complementary disciplines. Two papers published in *Advances in Physiology Education*, a journal of the American Physiological Society, call for changes in medical education to do so, with the aim of closing the knowledge gap between engineers and physicians [11,12].

### Recommendation 2: Education

Medicine has clumsily entered its digital age via the back door: vast and costly electronic medical records systems have been implemented largely without careful and planned consideration for their impact on the entire health care system, including education, practice, workflows, and research. Education and practice systems have not taken this new digitized world into full account, and consequences include students who are unprepared for their digital futures, very unhappy physicians stuck behind computer screens selecting seemingly endless items in reams of dropdown lists, and the unconscious loss of many opportunities for improvements in practice and research. It is time, even if a bit tardy and somewhat less than proactive, to acknowledge and address this transition of medicine from paper to computer, from opinion and experience to evidence, and from memory to search engines.

Previous reports have demonstrated a deficit in knowledge of clinicians and even clinician scientists relating to statistical methods and their applications to clinical data, including the data used in practice [13,14]. There appears to be little knowledge of, and seemingly even less interest in, these increasingly critical issues among most physicians. This unacceptable awareness and training gap has prompted updates internationally in medical curricula in order to include additional instruction in and exposure to statistical applications and epidemiology. However, there are inadequate numbers of physician educators who are equipped with the knowledge in informatics and data science required to provide even the most basic and essential insights to junior trainees [15].

The questions then consist of who, when, how, and what do we train? Training should focus on two groups of medical trainees: medical students and residents. If we accept that over the next half century there will likely be an increasing need for hybrid skills of this nature, then there is a strong case for inclusion of data science in the core curriculum in medical school and during residency training [16]. An introduction to the use of digital health records for research may provide a foundation to be able to contribute to knowledge discovery regardless of the career path medical students and residents eventually choose. This should then be followed by optional courses, preferably with practical research (eg, summer courses or internships), to further develop these skills for those particularly interested. This latter group is likely to form the core of future educators in this area.

Interested medical students and residents would benefit from educational opportunities that foster cross-disciplinary working relationships. One such experience might be the participation in the datathons that we have previously used in our own work to encourage collaboration [17]. The resources and insights generated during such events may be stored and used in ongoing collaborations and may be continually updated to provide greater scientific rigor and insight. A final suggestion is represented by online platforms and communities, where physicians and data scientists could interact, discuss clinically relevant questions, and share repositories of code and worked examples.

Perhaps most important, creating a medical culture that is aware of and respectful of the importance and potential power of data for supporting and improving both practice and research may be the most important and ultimately effective element. It is desirable that each participant in the clinical process realizes and understands their role in the overall system of providing reliable and robust data that they and others will subsequently use in improving care.

### Recommendation 3: Rethinking Academic Incentives

The education of medical trainees in data analysis methods and data scientist trainees in the particular domain issues of clinical practice and data would be a primer for future collaboration between the two groups later in their careers. Such cooperation could be fostered by policies on the part of academic journals that encourage joint submissions from clinicians and data scientists. The perceived “publish or perish” culture of academic medicine has not much changed for the past 30 years [18] and has led to all manner of trivialities: publishing on obscure or irrelevant results with minimal clinical or research importance; sectioning results into multiple manuscripts across numerous journals; and competing for data sets (or, at least, a lack of transparency and sharing of the same). While not a panacea for these ills, equal authorship may ease some of the barriers to cooperation between researchers and perhaps result in higher-quality publications. Big data lends itself better to collaboration than to separation—multiple studies can be run on the same dataset for different purposes. For instance, an emergency department data set from Hong Kong was used both to identify populations who are sensitive to extreme weather, *and* to develop a long-term forecast for ambulance demand through 2036 [19].

We predict that the continued success of such collaborative models will require the recognition of equal authorship as on par with first/last authorship as currently used by journals, universities, and funding agencies. We argue that the impact of the paper is more important than the individual contributions, provided the authors will testify they each had a meaningful role to play in the development of the final publication or proposal. Equal authorship may not end, but could temper, current publishing strategies where multiple, smaller, less impactful papers are released so as to procure first/last authorship for all members of the group—currently important for their curricula vitae and academic standing. A next step would be to give credit for the use of data by counterparties—how better to validate one’s cohort than to have other researchers use the same data for their own related (or unrelated) research questions? Any conclusions reached by the latter should in part be attributed to the former for their role in generating and collating a high-fidelity data set, and that role also recognized in academic circles. These measures would encourage investment in big data infrastructure while also improving the quality of “big data” conclusions.

### Recommendation 4: Funding

Fostering research in a specific field and encouraging collaboration between fields through funding is not new. Almost 20 years ago, the US National Institutes of Health (NIH) convened a Director’s Panel on Clinical Research to address the decline in physician investigators applying for clinical research grants. The panel proposed a series of recommendations to increase the funding opportunities for clinical researchers [20]. We suggest implementing many of the same methods today to encourage closer collaboration between clinicians and data scientists through funding and incentives.

NIH-funded research programs are classified using activity codes, with, for example, codes from the R series corresponding to research grants (eg, R01, R13) and from the K series corresponding to careers development awards. We suggest creating a K award category for clinicians engaged in data science similar to a K23 grant for clinicians that the Director’s Panel on Clinical Research proposed. Providing a unique K grant for data scientists would provide increased funding opportunities, since K awards typically fund 40% of applicants, unlike the R0 awards, which are more competitive and funded at a much lower rate, usually 10%. This support is critical to foster data science investigators during the vulnerable early period in their career.

By restructuring study sections and adding study sections across all the institutes exclusive to secondary analysis, grants would increase the funding available for data science proposals that may not otherwise obtain funding in the current structure. Having a study section specific to secondary analysis to score data science proposals would ensure that at least 10% of the data science proposals are funded (and avoid competing against primary analysis proposals, which might receive preferential scoring). In addition, mandating a health care provider as part of the proposal team would encourage the clinical impetus behind the proposal.

Another example of the US federal government increasing access to funding is the US Department of Veterans Affairs’ Big Data Scientist Training Enhancement Program, which has been adopted by 6 pilot centers [21]. The program supports data scientists working directly with clinician scientists on-site at a hospital.

## Conclusion

Better use of clinical data has the potential to address a number of important, problematic, and unresolved issues in the health care system. These include high, and perhaps excessive, costs; unnecessary and undesirable practice variation; the improvement of digital workflows; the universal implementation of a reasonable, reliable, and usable version of evidence-based medicine; the introduction of personalized and precision medicine; quality; safety; effective communication; efficient care coordination; and the introduction of data-driven and -supported clinical decision making. However, the introduction of this kind of revolution into health care inevitably involves crossing disciplinary boundaries in a way that requires cooperation and collaboration among a frankly diverse group of experts in order to optimize the combined output of these contributors. The formation of such teams requires that each team member be more educated in the issues involved outside of their own comfort areas. As a primarily medically oriented group, we focus on the impact on medical training, but the principles relate to those in nonmedical areas who need to become sufficiently educated in clinical matters to contribute optimally to the grand scheme. For example, how can current advanced analytic techniques such as machine learning be best applied to both clinical research and practice problems? Clearly, specific kinds of clinical-technical collaborations will be required to guide these kinds of processes and projects to fruition.

In this paper, we have attempted to portray not just the problems, but also potential solutions, or at least beginning approaches to solutions, for the situation in which we find ourselves. This situation involves a costly, complex, and massive health care system that can well bear improvement, and a growing mound of underutilized data that is accumulating as a result of the accelerating digitization of medical care.

Clinicians should not feel like interchangeable cogs entering reams of data blindly into a vast black hole of no return; data scientists should not be discovering new knowledge and developing predictive algorithms isolated from the domain experts. Rather, all should see themselves as diversely necessary components of a truly functional clinical data system that works toward providing excellent care to individuals and populations while working to improve all facets of that care.

## Take-Home Messages

1. Fundamental quality, safety, and cost problems have not been resolved by the increasing digitization of health care.

2. This digitization has progressed alongside the presence of a persistent divide between clinicians, the domain experts, and the technical experts, such as data scientists.

3. The divide can be narrowed by creating a culture of collaboration between these two disciplines, exemplified by events such as datathons.

4. However, in order to more fully and meaningfully bridge the divide, the infrastructure of medical education, publication, and funding processes must evolve to support and enhance a learning health care system.

## Abbreviations

AHRQ: Agency for Healthcare Research and Quality
NIH: National Institutes of Health
